# Ni(II) and Pb(II) Removal Using Bacterial Cellulose Membranes

**DOI:** 10.3390/polym15183684

**Published:** 2023-09-07

**Authors:** Francisco de Borja Ojembarrena, Sergio García, Noemi Merayo, Angeles Blanco, Carlos Negro

**Affiliations:** 1 Department of Chemical Engineering and Materials, University Complutense of Madrid, Avda. Complutense s/n, 28040 Madrid, Spain; sergga06@ucm.es (S.G.);; 2 Department of Mechanical, Chemical and Industrial Design Engineering, High School of Technical Industrial and Design Engineering (ETSIDI), Universidad Politécnica de Madrid, Ronda de Valencia 3, 28012 Madrid, Spain; n.merayo@upm.es

**Keywords:** heavy metal adsorption, nanocellulose, bacterial cellulose, surface crystallization, kinetic modelling, isotherm, adsorption crystallization mechanism

## Abstract

Bacterial cellulose (BC) is a highly crystalline nanosized material with a high number of active groups. This study focuses on the synthesis of BC membranes through fermentation, their characterization and application to remove Ni(II) and Pb(II) from wastewater by adsorption under different conditions. Four-day-grown BC membranes form three-dimensional nanofibril networks with a pH of 6.3 and a high cationic demand (52.5 μeq·g^−1^). The pseudo-second-order kinetic model and the Sips isotherm model best describe the adsorption of both metals. The intraparticle diffusion model of Ni(II) revealed a three-step mechanism of adsorption-plateau-adsorption, while Pb(II) adsorption followed a typical reducing-slope trend up to saturation. The highest removal of Ni(II) and Pb(II) was obtained at pH 4 with a BC dosage of 400 mg·L^−1^. The maximum adsorption capacities were 28.18 mg·g^−1^ and 8.49 mg·g^−1^ for Ni(II) and Pb(II), respectively, involving the total coverage of the material active sites. Thermodynamically, Ni(II) adsorption was exothermic, and Pb(II) was endothermic. The obtained values of sorption heat, activation and Gibbs’ energy depicted a physisorption process. Ni(II) removal mechanism was ruled by crystallization on the metals adsorbed on the BC active groups, while Pb(II) was driven by the adsorption process, as shown by TEM images of the spent material.

## 1. Introduction

The removal of heavy metals from effluents, even when they are present at low concentrations, is essential for complying with regulatory standards in order to protect human health, preserve the ecosystems and ensure safe drinking water. It is a crucial step to achieve sustainable processes. Therefore, great efforts have been carried out in recent decades to reduce the environmental impact of the most problematic metals, such as mercury, lead, chromium, arsenic, and copper, among others. However, some industrial sectors highly depend on these metals and are expected to have a significant increase in their demand. For example, the transition towards electric vehicles and renewable energy systems relies heavily on advancements in battery technology. The battery sector consumes more than 80% of total lead for industrial applications to produce the traditional lead-acid batteries for the automobile sector, uninterruptible power supply, and backup power systems. The study by Yang et al. revealed that in China, the lead-battery industry discharges to the water around 0.2 kg of lead per kVAh of battery power [1]. This value means the spillage of 1% of the total lead usage for lead-acid batteries in the country suppose up to 2010 tons of uncontrolled discharge of lead to natural water courses in 2017. However, lead-acid batteries are gradually being replaced in certain applications due to their lower energy density and limited lifespan. Nickel and lithium are being used in modern batteries because of their higher energy density, light weight and faster charging [2]. In accordance with the global electric vehicles (EV) outlook of the International Energy Agency, the EV battery sector will have an exponential growth of around 8% in 2023 [3]. This report also reflects the importance of nickel usage for EV batteries, which has suffered a six-fold increase from 2017 to 2022. Nickel has become a metal of high relevance, and its annual demand has surpassed the annual supply for four years in the noted interval. The presence of nickel and lead in the environment would provoke severe consequences. These heavy metals are toxic and persistent, and exposure to them, even in small amounts, can cause various health issues, including neurological damage, kidney damage, respiratory problems, developmental issues in children, and even cancer. These metals can easily enter the food chain, accumulate in living organisms, and eventually reach humans through the consumption of contaminated food and water. By removing nickel and lead from discharged effluents, the risks of exposure can be minimized and the overall burden on ecosystems and human populations would be considerably reduced [4]. Due to these harmful affections, their discharge limits are low. Nickel maximum allowable discharge values in Spain vary from 0.5 to 10 mg·L^−1^, while the lead ones can be found in the interval between 0.5 and 3 mg·L^−1^ [5]. The rising global concern about water contamination indicates that the mentioned limits would be strictly reduced soon with the independence of the country. Furthermore, in the case of nickel, novel recovery treatments are essential to improve the sustainability and the circular economy of the battery sector. This will be a critical factor to succeed in the coverage of the nickel demand when circulation restrictions for nickel as a strategic material are enforced. Overall, the need for new solutions to further prevent the discharge of nickel and lead is essential for the future. 

The removal of nickel and lead from industrial effluents depends on the initial concentration and typically requires a combination of technologies and best practices. Physical-chemical treatments are highly efficient and robust and can be enhanced by pH optimization. Some examples are chemical precipitation, coagulation–flocculation, adsorption, and its advanced variations, like electrocoagulation, membrane separation and ion exchange [6,7]. At high concentrations, precipitation is commonly used, while adsorption is preferred at lower concentrations [8,9]. Adsorption processes at industrial scale are usually performed using activated carbons and zeolites [10]. However, in recent decades, a significant research effort has been carried out to locate greener adsorbents focusing on their efficiency, specificity, resistance, recovery, biodegradability and sustainability [11,12]. Nanomaterials such as nanocellulose have shown great potential as bioadsorbents for heavy metals [9,13,14]. 

Nanocellulose products can be produced from renewable natural resources or from lignocellulosic wastes, contributing to a circular economy [15]. They are non-toxic and have unique adsorption properties due to their high surface area and abundance of active groups that are easily functionalized [16]. Although promising results have been achieved with fibrillated cellulose and cellulose nanocrystals for the adsorption of heavy metals, their small sizes make for the difficult separation of these products from the treated water at the industrial scale. In fact, some authors have applied surface magnetization techniques to cellulose nanocrystals to facilitate the separation during their application [17]. 

On the other hand, bacterial cellulose (BC) is an extracellular cellulose produced as a film by static culture of some Gram-negative species of bacteria of the genus *Acetobacter* [18]. These bacteria produce pure cellulose nanofibers from 20 to 100 nm in diameter that are largely crystalline and with a high polymerization degree and purity [19]. The nanofibers are entangled in a three-dimensional porous network that forms a film or membrane that may be a few centimeters thick, depending on the culture time [20]. These microstructure membranes present various advantageous properties, such as high tensile strength, elasticity, microporosity and high surface area for their application as adsorbent, and they can also be easily removed from the water after the treatment. The BC worldwide demand has increased in recent years, especially in the food industry, where it is applied as a jelly raw material, as well as a sauce and drink additive and garnish to dishes [21]. 

The BC market reached USD 207 million and is expected to surpass USD 700 million. The industrial production of BC is carried out in static cultures (tray production) or in agitated cultures, obtaining large BC pellicles or BC slurries in each case [21]. In order to improve their efficiency, nanocellulose has been modified, functionalized and combined with other organic and inorganic materials, forming different nanocomposites [22] and nonwoven adsorbents [23]. BC nanocomposites have found a variety of applications in several areas of higher added value than wastewater treatments [22]. 

In the case of lead adsorptions, the best results are achieved by the surface modification of BC by carboxymethylation [24], polyethyleneimine [25], diethylenetriamine [24,26,27] and amidoximation [27]. Carboxymethylated BC improved the performance of BC by 30% (12.6 mg·g^−1^) in the case of Cu(II) and 167% (60.4 mg·g^−1^) for Pb(II) at an optimized pH of 4.5. Diethylenetriamine BC was more efficient, achieving a maximum adsorption capacity of Cu(II) and Pb(II) of 63.09 and 87.41 mg·g^−1^, respectively. With polyethilenimine BC, the maximum adsorption capacity of Cu(II) and Pb(II) was found to be 148 and 141 mg·g^−1^, respectively, which was higher than that of unmodified BC and other modified BC reported. All of the above closely followed the pseudo-second-order kinetic model, indicating the importance of chemical adsorption in the process. In the case of amidoximated BC, maximum adsorption was found at pH 5 with 84 and 67 mg·g^−1^, for Cu(II) and Pb(II), respectively. 

The BC adsorption of nickel is not well known. Mohite and Patil have studied the potential of BC as a bioadsorbent. In this case, BC was produced under shaking conditions and in single tests with various heavy metals, dyes and bovine serum albumin [28]. The removal of lead, nickel and cadmium were compared in this study, revealing a more efficient interaction of the cellulose with lead (82% Pb(II), 41% Ca(II) and 33% Ni(II) removals). 

Although culture time is an essential parameter affecting the depth and dried mass of the BC membranes, the kinetics of BC production are relevant. This parameter is not commonly covered by articles related to their application as bioadsorbent. To enhance the viability of the BC application, obtaining an optimal incubation time is essential so the culture medium with bacteria can be seeded again and keep a stable batch production. In this study, an evaluation of the membrane production kinetics to obtain membranes with similar conditions as well as a larger amount of BC to be tested as adsorbent, will be performed.

Although the state of the art shows the potential of BC as a bioadsorbent, there is a lack of knowledge on the optimal operating conditions and on the removal mechanism of lead and nickel onto unmodified BC. The application and optimization of the main parameters for the usage of this material in wastewater treatments for heavy metal removal is still a research challenge and is supposed to advance in the search for novel alternatives and sustainable nanomaterials for this purpose. Furthermore, the study and characterization of both raw and spent BC membranes after adsorption are still not deeply covered in the bibliography. This study will show critical information about the interaction between the BC and the metals and the evaluation of the material before and after will provide useful information about the main mechanisms of removal of the studied metals. Therefore, the objective of this study is to generate new knowledge on the efficiency, optimal conditions, material characterization, and BC membrane mechanisms for the removal of nickel and lead from wastewater.

## 2. Materials and Methods

### 2.1. Materials

The BC was synthetized by the pure bacterial strain of *Komagataeibacter sucrofermentans* DSM 15973, which was supplied by the German Collection of Microorganisms and Cell Cultures (DSMZ). Glucose, fructose, yeast extract, peptone, disodium hydrogen phosphate (Na_2_HPO_4_), citric acid (C_6_H_8_O_7_), sodium chloride (NaCl), potassium chloride (KCl), calcium chloride dihydrate (CaCl_2_·2H_2_O), sodium bicarbonate (NaHCO_3_) nickel chloride hexahydrate (NiCl_2_·6H_2_O) and lead nitrate (Pb(NO_3_)_2_) of analytical grade, as well as standard solutions of each metal to perform the calibration of the device were supplied by Sigma Aldrich (St. Louis, MO, USA). The concentration of nickel and lead during the experiments was measured through spectrophotometry by means of the visocolor ECO Nickel and Nanocolor Lead 5 reagent kits, respectively, supplied by Macherey Nagel (Düren, Germany). 

### 2.2. BC Synthesis

The bacterial culture and the BC membrane synthesis procedures were carried out as indicated by Santos et al. [29], but in this case, the initial bacterial strain was *Komagataeibacter sucrofermentans* DSM 15973. The necessary media and solutions were Hestring-Schramm media, both glucose- or fructose-based (from now, HS glucose and HS fructose), and Ringer’s solution, which were synthetized following the same compositions and concentrations as indicated by Santos et al. [29]. The employed experimental protocol is synthetized below.

#### 2.2.1. Bacterial Growth

The applied bacterial growth medium was a thermally sterilized HS glucose medium. Pure *Komagataeibacter sucrofermentans* DSM 15973 colonies were seeded into sterilized lab beakers filled with HS glucose medium (100 mL), which were then closed and stored under static conditions for 4 days at 37 °C. *K. sucrofermentans* bacteria grew in static conditions, in the upper part of the medium, in the liquid-gas interface between the medium and the inner air. There, the bacteria can simultaneously generate BC membranes while the population grows. Once finished, the disc-shaped BC membrane can be separated from the medium in a laminar flow cabinet, and each membrane was cut into four smaller pieces. The membrane pieces submerged in the medium were agitated in a type of “vortex” multireax (Heidolph, Schwabach, Germany) for 30 min to separate the bacteria supported on the membrane surface. The filtered liquid was then centrifuged. The bottom pellets were separated from the liquid and washed with Ringer’s solution up to the same volume of the previously added HS glucose. After stirring and centrifugation, the pellet is placed into a small Eppendorf tube and filled up with Ringer’s solution. The suspension was analyzed through a UV–Vis spectrophotometer, where the optical density at λ = 600 nm was obtained and, then adjusted to 0.59 to 0.64, which corresponds to 3 to 4 in MacFarland standards, being a concentration of about 1 × 10^9^ cells·mL^−1^ [30,31]. 

#### 2.2.2. Production of Bacterial Cellulose Membranes

A volume of 250 μL of the previously optical density-adjusted suspension was added to sterilized HS fructose, which is the BC production medium, following the same culture procedure as indicated before. The culture time was up to 15 days while analyzing the BC membrane growth curve. Once the BC fermentation was finished, it was removed by filtration from the production medium and washed thoroughly with Ringer’s buffer through agitation in the multireax device. The washed membranes were then treated at 90 °C with 1% of NaOH for 30 min for a final disinfection step. The disinfected BC was washed with distilled water to remove the excess NaOH until the pH of the washing water was stable. 

### 2.3. BC Characterization

The calibration curve of BC growth was evaluated under various fermentation times to predict the growth of the material and the possible final weight of the dried product at the end of the fermentation process. BC membranes were characterized considering the relevant physical-chemical properties that determine their performance as adsorbents. BC consistency was carried out as indicated by Balea et al. [32] by calculating the dry mass content. The measurement of the pH zero charge of the membranes (pH_zc_) was performed as established by Hosseini Talari et al. [33] to evaluate the pH value where the surface of the BC suffers a charge neutralization. Basically, the BC pellicles are placed in distilled water whose pH has been increased or reduced with NaOH or HCl solutions, respectively, and after a certain contact time, the pellicle is removed from the solution, and the pH is measured again. To evaluate the pH_zc_, the difference between the initial and final pH (ΔpH) is plotted versus the initial pH, so the point where ΔpH is equal to zero can be easily determined graphically or by mathematical adjustment of the data. The cationic demand of the material was determined following the protocol described by Ojembarrena et al. to measure the anionic demand of hairy cellulose nanocrystals [34], with the modification of using cationic N-polydiallyldimethylammonium chloride (polyDADMAC) as titration reagent which reacts with the anionic surface of BC until reaching the isoelectric point. Transmission electronic microscopy (TEM) images of the initial and spent cellulosic material with nickel and lead onto its surface were taken with a JEM-1400 plus (Jeol, Peabody, MA, USA) Electronic Microscope (×1000 magnification) for an accurate analysis of the surface morphology of the material and determination of the removal mechanism. 

### 2.4. Batch Adsorption Tests

#### 2.4.1. Experimental Methodology: Equilibrium Experiments

The equilibrium tests were carried out at several pHs, BC dosages and initial metal concentrations. These experiments were performed in batches in triplicate. The samples of synthetic water contaminated with Ni(II) or Pb(II) were introduced in Falcon tubes filled up to 25 mL under the selected conditions for each sample with the adequate dosage of BC. These tubes were stirred for three hours in the multireax device and filtered at 0.45 µm after the adsorption test had finished. The treated and blank filtered water samples were analyzed to obtain the removal yields and adsorption capacities of the contaminants at the equilibrium at each analyzed condition. The experimental conditions varied depending on the analyzed parameter:

- pH optimization. Fixed conditions: 25 mL of sample volume, 5 mg·L^−1^ of initial metal concentration and 400 mg·L^−1^ of dried BC dosage; variable conditions: operating pH 2–8;
- Dosage of BC optimization. Fixed conditions: 25 mL of sample volume, 5 mg·L^−1^ of initial metal concentration and operating pH of 4; variable conditions: dried BC dosage 10–400 mg·L^−1^;
- The initial concentration of metal optimization (isotherm experiments). Fixed conditions: 25 mL of sample volume, operating pH of 4 and 400 mg·L^−1^ of dried BC dosage; variable conditions: initial metal concentration 1.5–150 mg·L^−1^ for Pb(II)) and 1.5–175 mg·L^−1^ for Ni(II).

#### 2.4.2. Experimental Methodology: Equilibrium Experiments

The kinetic experiments were used to analyze the effect of contact time and temperature in the operation of BC with Pb(II) and Ni(II). These tests were carried out in lab glass beakers filled with 100 mL of the synthetic waters under the previously optimized operating parameters. Different samples were taken and filtered at 0.45 µm with syringe filters at different contact times, up to 3 h. As in the equilibrium tests, soluble nickel and lead were measured to determine the efficiency of the process and to achieve the different points to perform the posterior kinetic study. 

The operation conditions applied during these experiments were:

- Temperature effect. Fixed conditions: 25 mL of sample volume, 1.5 mg·L^−1^ of initial metal concentration, operation pH of 4; 400 mg·L^−1^ of dried BC dosage; variable conditions: operating temperature 25–50 °C, contact time 1–180 min.

#### 2.4.3. Experimental Methodology: Determination of Metal Concentration

The determination of nickel and lead was performed by using an Aquamate UV–Vxis spectrophotometer (Thermo Scientific, Waltham, MA, USA), which was previously calibrated with the corresponding measurement kits and standard solutions of each metal. The selected wavelength was the top absorbance peak for both nickel (470 nm) and lead (520 nm) recommended by the supplier of the kits, which is based on the German standard DIN 38 402-A51, DIN 32645 and DIN ISO 5725 [35,36,37].

#### 2.4.4. Data Analysis

The resulting data were evaluated to reach the operating conditions that were associated with the maximum removal of the selected metals. On the one hand, the BC growth kinetics, the equilibrium data from variable pH and dosage adsorption tests of nickel and lead were fitted to logarithmic, linear, polynomial, or potential equations, and the best-fitting equations of each experimental data were plotted. The analyzed data were evaluated through OriginPro 9.6 2019 data analysis software. On the other hand, kinetic and isotherm data were adjusted to the selected models by fitting these experimental results to their linear equations. These final adjustments were analyzed as indicated in previous works by Ojembarrena et al. [34,38]. The chosen kinetic models are pseudo-first-order (from now PFO), pseudo-second-order (PSO), Elovich and intraparticle diffusion (ID) models. Their corresponding equations can be seen in Table S1 in the Supplementary Material. 

Isotherm data were fitted to Langmuir, Freundlich and Sips models to evaluate the adsorption mechanism, while through the Temkin and Dubinin–Raduskevich (D-R) equations’ parameters, the values of heat of sorption and mean free energy of Gibbs were obtained. All selected kinetic and isotherm models were initially fitted to linear adjustments. After that, a posterior non-linear fitting through Excel provided a more accurate value of the parameters by minimization of the residual sum of squares (RSS). The selected models and the equations for the isotherm models are shown in Table S2 in the Supplementary Material. The equations needed to find the heat of sorption parameter from Temkin isotherm (b_T_) and the mean free energy of Gibbs from D-R isotherm (E_DR_) are also indicated in Table S2, found in the Supplementary Material.

The data analysis covered the temperature effect study through the analysis of the obtained kinetic constants from PFO, PSO and ID by means of the Arrhenius’ Equation (1).

(1) $$\ln\left(k\right)=-\frac{E_{A}\;\left(J\cdot {\mathrm{mol}}^{-1}\right)}{R\;\left(J\cdot {\mathrm{mol}}^{-1}\cdot K^{-1}\right)\cdot T\left(K\right)}+\ln\left(k_{0}\right)$$

where *R* is the ideal gas constant, *T* is the temperature and *E_A_* is the energy of activation of the process (in SI units). 

The obtained values of energy of activation, heat of sorption and mean free energy can be then interpreted to know if the energy level of the metal adsorption is in the field of physical interactions (or physisorption) or if there is a higher amount of energy exchange involved in the process, typical from chemical sorption. 

## 3. Results and Discussion

### 3.1. BC Characterization

The first characterization of the BC membranes was their consistency. The produced BC membranes showed a 0.96% of cellulose-dried mass at 60 °C. This value is higher but in the same order of magnitude as the dry/wet weight ratio of the BC pellicles obtained by Budhiono et al. [39], which ranged 0.4–0.7 g of dry BC mass per 70–90 g of wet membrane. The difference can be attributed to the use of HS fructose production medium used in this experiment, whose origin was synthetic, and the components were precisely dosed, in comparison to the one used by Budhiono et al., which was based on fermented coconut water with the addition of glucose and nitrogenated organic compounds. 

**Figure 1:**
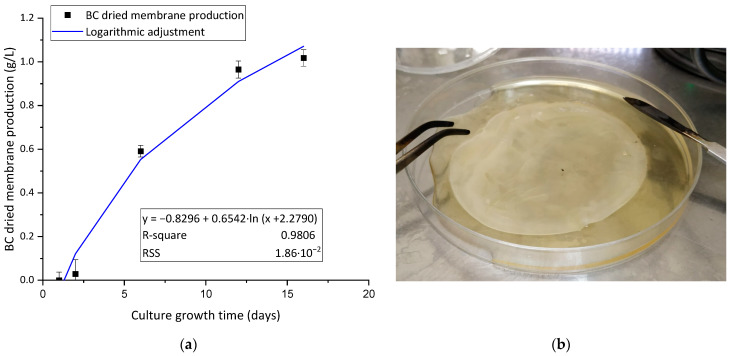
(**a**) BC membrane growth kinetics during different bacteria culture times (black squares) and logarithmic adjustment (blue line); (**b**) image of 4 days of membrane growth.

The growth curve of the BC membranes along the culture time was evaluated. The obtained results are plotted in Figure 1a. The kinetic curve indicates that low membrane production is seen before the third day of growth due to the need for adaptation of the bacteria to the fructose medium. The initial bacterial seed is taken from a HS glucose growth medium and needs to modify its metabolic pathways to adapt to fructose and produce BC. This adaptation of the bacterial metabolic pathways to the carbon source was previously established by Santos et al. [29]. The curve shows that the experimental data could be well adjusted to a modified-logarithmic fitting and indicates a lag time of about 2.28 days. The fitted curve shows a high slope at the beginning and a strong reduction in the production rate before the fifth day of production. This factor is critical, as it is important that the isolated cells from the production medium show the highest BC synthesis capacity and rate at the point the BC membrane is purified to allow their reintroduction in a new synthesis batch with fresh HS fructose. Long culture times provide membranes that grow in thickness but would not increase their area. In terms of applicability, this means further mass transfer limitations of metal ions from the bulk to the inner core of the BC membrane, which is not desirable and would probably reduce the mass adsorption efficiency of the membrane. For this reason, 4 days of culture was established as an adequate time to obtain stable membranes with mechanic strength for high-stirring conditions and easy to separate but at the same time maintaining a high mass-area ratio to enhance their adsorption capacity per unit of mass. This result is of main importance for the research and proposes a novel way to obtain a sustained production of BC by batches with similar conditions to allow a good reproducibility of the adsorption tests. The image seen in Figure 1b corresponds to 4 days of growth of the BC membrane. This synthesis route is key for the viability of the adsorbent production, as it provides a continuous way of synthetizing BC through consecutive batches without the need to generate new batches of bacteria from the growth medium (HS glucose). 

The adsorptive capacity and surface charge of the synthetized BC were analyzed by cationic demand titration. A total of 52.5 ± 3.2 μeq·g^−1^ was measured in the obtained BC material. This value shows that the produced BC membrane is covered by anionic charges onto its surface and that the cationic demand value corresponds to the maximum cationic charges that the membrane could attract and catch onto its surface. 

**Figure 2:**
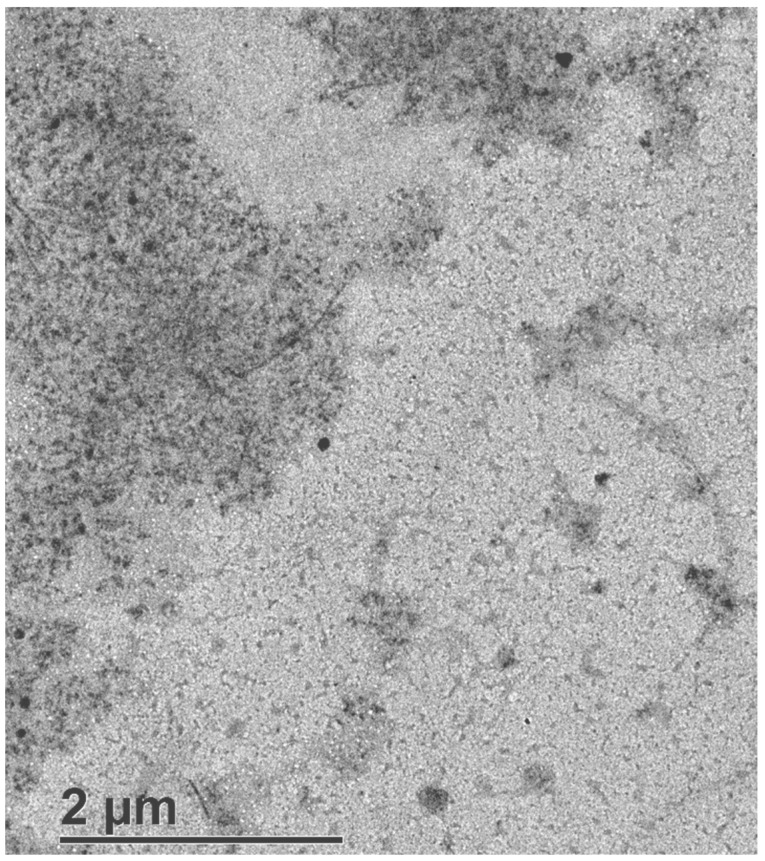
TEM image of raw BC membrane.

Raw BC was observed through TEM (Figure 2). Large networks of BC were identified on the left and upper areas of the image. This entangled network of BC nanofibrils was similar to what was previously reported by Usawattanakul et al. [40]. While the average size of the BC membrane network clusters lies in the order of micrometers, the mean diameter of the cellulose fibers that compose this matrix is in the order of a few nanometers. The darker dots that appear embedded inside the BC matrix can be associated with fragments or complete cells of dead bacteria that were not removed in the washing steps. This presence of wrapped cells in the dense BC matrix was also observed by Campano et al. in BC membranes produced using static culture conditions [41]. 

**Figure 3:**
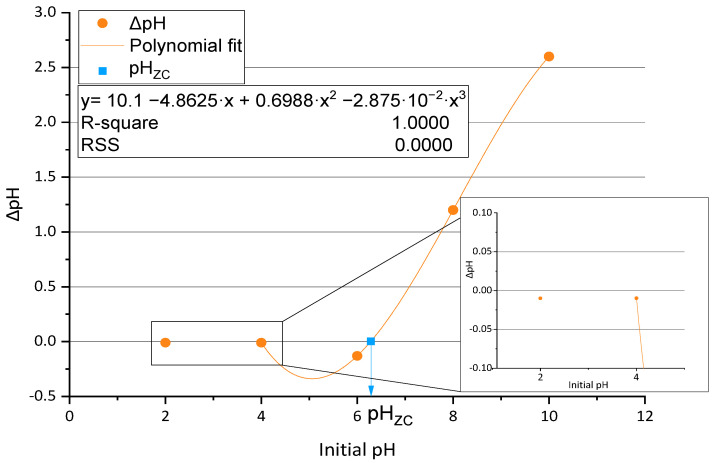
Experimental evolution and polynomic adjustment (orange line) of ΔpH with the initial pH (orange dots) and pH_zc_ value (blue square).

Once the surface morphology and the BC charge were analyzed, it was important to evaluate the change of the surface charge with the pH. The results of the pH_zc_ tests of the BC membranes are shown in Figure 3. The value of pH_zc_ was calculated by adjusting the experimental results to a polynomial fitting. The values of ΔpH between initial pH values of 2 and 6 were negative, being close to zero for initial pH values of 2 and 4. This fact indicates a low interaction between the material surface and the solutions in this last interval. The trend varies between pH 6 and pH 8, where the ΔpH becomes higher as pH increases. After the polynomic fitting is performed, it becomes clear that the solution of this equation, when ΔpH is zero between 6 and 8, will show the precise value of the pH_zc_, which in this case corresponds to a value of pH_zc_ of 6.31. The application of BC below this value will provide a negative surface charge while being positively charged when added to more alkali solutions. 

### 3.2. Adsorption Tests Experimental Results

#### 3.2.1. pH Optimization

**Figure 4:**
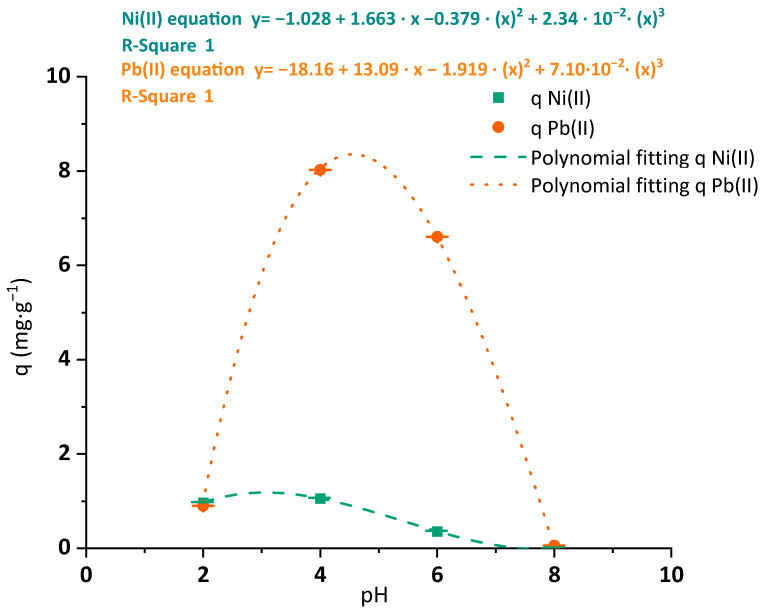
Maximum adsorption capacities of Ni(II) (green squares) and Pb(II) (orange dots) and polynomic adjustment (blue dashed and orange dotted lines, respectively) onto BC membranes under varied treatment pH values (2–8). Tested conditions: 25 mL of sample volume, 5 mg·L^−1^ of initial metal concentration and 400 mg·L^−1^ of a dried BC dosage.

The first operating condition to be evaluated was the pH. This parameter is a key factor, as expected by the results of the pH_zc_ analysis. The evolution of the maximum adsorption capacities achieved through batch experiments of both Ni(II) and Pb(II) are shown in Figure 4. Although the trend was similar in both cases, BC has a clearly different metal affinity for Ni(II) and Pb(II), showing an eightfold higher adsorption capacity of Pb(II) under the tested conditions rather than nickel one. This affinity was previously mentioned by Mohite and Patil and Attar et al. [28,42]. The maximum experimental adsorption capacity of both metals was reached at pH 4. The calculated pHs to achieve the highest removal for Ni(II) and Pb(II) through polynomic fitting were 3.05 and 4.57. Thus, the experimental condition of pH during the rest of the batch adsorption tests for each metal was maintained at pH 4. The maximum adsorption capacity of Pb(II) in BC membranes, close to 10 mg·g^−1^, depicts a high bonding affinity even under low concentrations of metal (just 5 mg·L^−1^ in this experiment) between pH values of 4 and 6, reflecting an efficient performance of the material. Chen et al. also revealed a stronger adsorption capacity of this metal in pH values between 4 and 6 than under pH 2 conditions, but no results of adsorption experiments over pH 6 values were reported [24]. 

The adsorption capacities of both metals fell to zero when the pH values overpassed the pH_zc_ of the BC membrane (pH_zc_ 6.30). This fact is associated with the modified surface charge of the material explained in the characterization. The cationic behavior of the BC surface with pH values higher than pH_zc_ would cause the repulsion of other cations present in the solution, minimizing its adsorption capability of Ni(II) and Pb(II).

#### 3.2.2. Dosage Optimization

**Figure 5:**
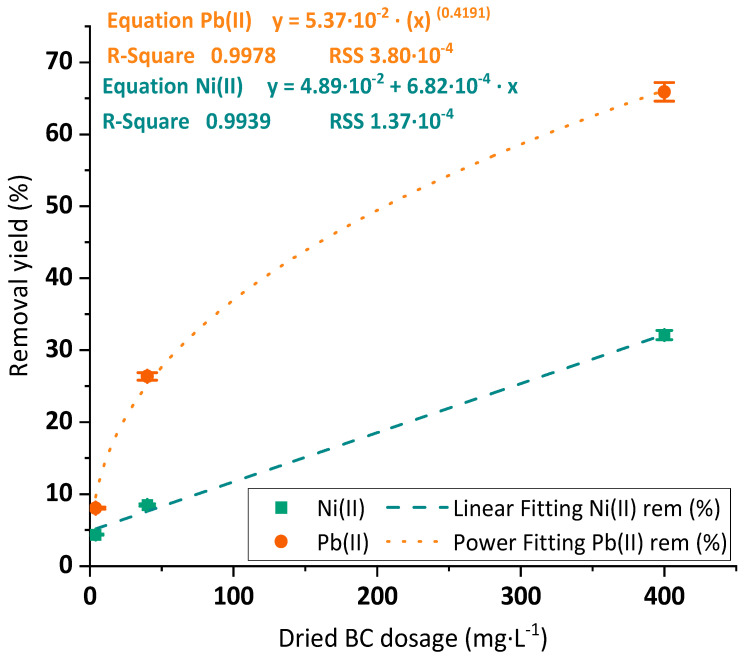
Removal yields of Ni(II) (green squares) and Pb(II) (orange dots) and linear and power adjustments (blue dashed and orange dotted lines, respectively) onto BC membranes under varied BC dosage values (4–400 mg·L^−1^). Tested conditions: 25 mL of sample volume, 5 mg·L^−1^ of initial metal concentration and pH 4.

The removal yields of Ni(II) and Pb(II) by applying an increasing BC dosage were determined. The experimental results of the removal yield of both Ni(II) and Pb(II) while rising adsorbent dosage and their corresponding adjustment to different mathematic equations can be observed in Figure 5. The represented curves indicate a larger adsorption removal of Pb(II) (65.9%) than the one of Ni(II) (32.1%) at the tested conditions of low metal concentrations under the highest dosages. As in the previously evaluated condition of pH, this fact suggests an easier interaction of the BC surface groups with the dissolved Pb(II) than in the case of Ni(II). The maximum adsorption capacities of Pb(II) and Ni(II) in these experiments were reached under the lowest dosages (4 mg·L^−1^) and corresponded to 25.90 mg·g^−1^ and 16.07 mg·L^−1^. These results lay in the order of magnitude of the ones mentioned by Chen et al. for raw BC with Cu(II) and Pb(II) by applying similar dosages [24]. 

#### 3.2.3. Kinetic Analysis

**Figure 6:**
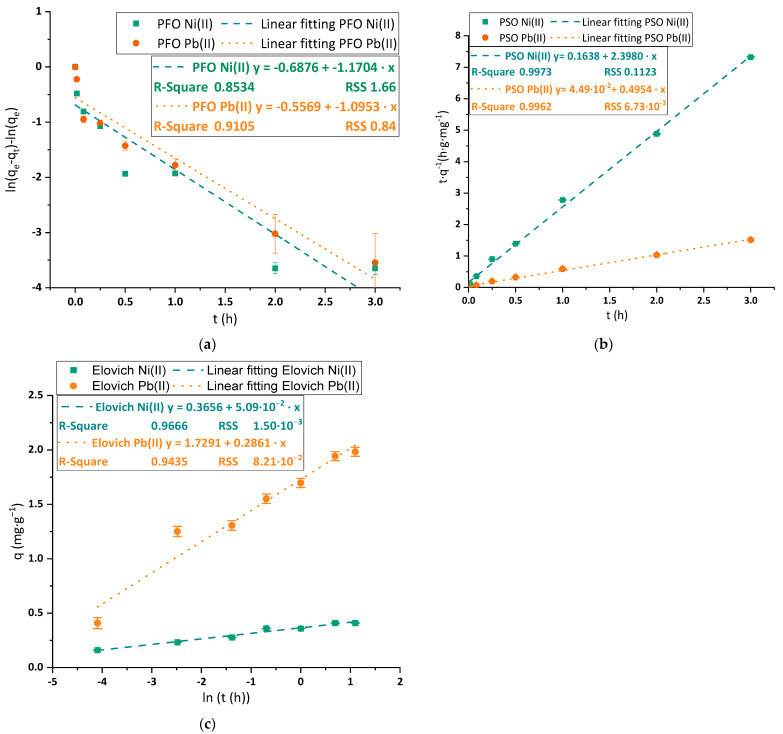
Linear adjustment of the selected kinetic models of (**a**) PFO; (**b**) PSO; and (**c**) Elovich equations to experimental data for Ni(II) (green squares, fitting in green dashed line) and Pb(II) (orange dots, fitting in orange dotted line). Tested conditions: 100 mL of sample volume, 1.5 mg·L^−1^ of initial metal concentration and pH 4.

The evaluation of the kinetics was performed by obtaining different experimental data from batch adsorption tests at different contact times of the synthetic solutions of the studied metals with BC membranes. The results were initially fitted to PFO, PSO and Elovich kinetic models to predict the adsorption behavior of the material with Pb(II) and Ni(II). The results of the adjustment of the previously said kinetic equations to Pb(II) and Ni(II) can be seen in Figure 6a–c. The kinetic constants associated with each model and metal are shown in Table S3. 

As indicated in Figure 6a, the PFO linear equation did not represent the kinetics of the adsorption process in any of the tested cases. However, the linear fittings show that PSO (Figure 6b) was the best-fitting model, reaching a R^2^ upper than 0.99 for both metals. The high correlation coefficient of PSO to reproduce the adsorption kinetics of different metals onto nanocelluloses has been reported in the past and could imply the presence of a chemisorption process. The PSO mechanism is explained by the need for two active sites for occupancy. This model also suggests that intraparticle diffusion would play a major role in the adsorption mechanism [43]. Such accuracy in the representation of the experimental data would enhance the posterior simulation or prediction of the results of the BC adsorption process with a view to further implementation of the material at a larger scale or treating a complex medium. The Elovich model (Figure 6c) showed a lower correlation coefficient than PSO but could slightly predict the adsorption kinetic data, especially in the case of Ni(II) (R^2^ = 0.9666). In fact, as indicated by the posterior non-linear analysis, this model provides similar but even lower RSS values than PSO, which suggests that the mechanism proposed in the Elovich model could also explain the removal process of each metal. This model describes the reduction of the adsorption rate with time caused by the surface coverage of the adsorbent [44]. 

**Figure 7:**
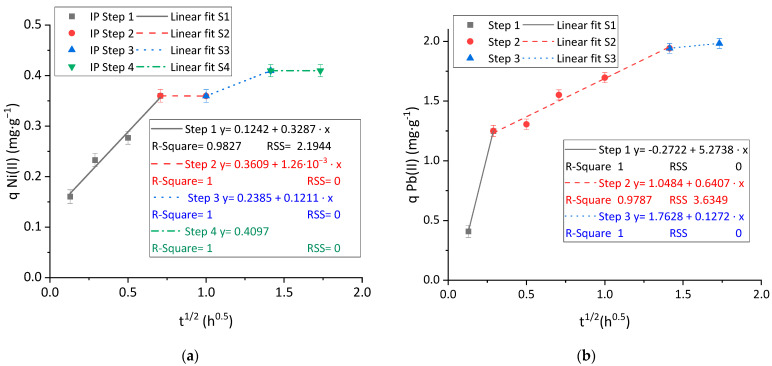
Linear adjustment of the ID kinetic model to experimental kinetic adsorption data of (**a**) Ni(II) and; (**b**) Pb(II). Tested conditions: 100 mL of sample volume, 1.5 mg·L^−1^ of initial metal concentration and pH 4.

The presence of intraparticle diffusion limitations in the mass transfer of the cations to the anionic surface of the material was demonstrated by the adjustment of the ID or Webber and Morris model to the observed steps in the kinetic adsorption curves. The results obtained for Ni(II) and Pb(II) are plotted in Figure 7a,b. 

The evolution of Ni(II) (Figure 7a) and Pb(II) (Figure 7b) is widely different. Firstly, the intercept of the first step, which represents the effect of external diffusion, is both far from the origin (around the third part of the maximum adsorption capacity) and positive in the case of Ni(II) adsorption, revealing a strong effect of boundary layer and, thus, a relevant external mass transfer limitation effect [45]. On the other hand, the first intraparticle step of Pb(II) adsorption crosses an intercept value closer to the origin and negative. The negative value is less typical than the positive values [45] and shows an intense interaction with the material, which is demonstrated by the high kinetic constant achieved (up to 16 times higher than the Ni(II) one).

The shape of the ID curves is different for both metals. The curve of adsorption of Ni(II) reveals the presence of intermediate plateau steps, where the adsorption rate is almost zero, between faster adsorption steps. This shape is quite uncommon in adsorption applications to wastewater treatment but has been previously reported in the application of nanocelluloses to hexavalent chromium removal [34,38] and could be associated with the presence of other phenomena that could happen sequentially to the adsorption process and which are involved in the metal removal. As an example of a combination of other mechanisms together with adsorption for nickel removal under slightly acidic conditions, Miller et al. proposed a combined surface precipitation and adsorption removal mechanism for nickel from acid mine drainage using limestone and hydrous ferric oxide [46]. On the other hand, the removal of Pb(II) shows a slope-reducing sequence of steps until reaching the saturation of the material, which is attributed to the rates of the diffusion steps of Pb^2+^ cations on their way to the active sites. In general terms, the adsorption of Pb(II) was faster and reached higher adsorption capacities than the case of Ni(II). 

#### 3.2.4. Temperature Effect

**Figure 8:**
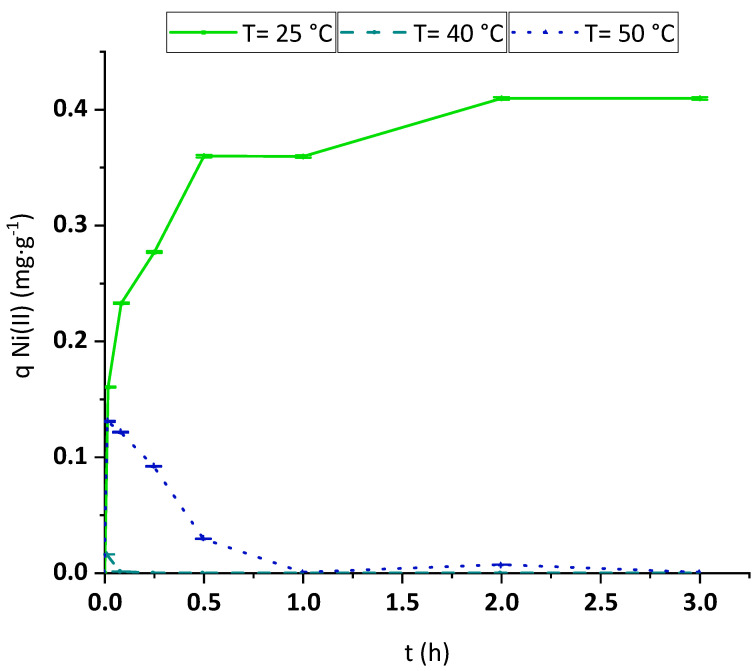
Evolution of adsorption capacity of Ni(II) onto BC in batch kinetic experiments at 25 °C (green continuous line), 40 °C (turquoise dashed line), and 50 °C (dark blue dotted line). Tested conditions: 100 mL of sample volume, 1.5 mg·L^−1^ of initial metal concentration and pH 4.

The temperature effect on the Ni(II) and Pb(II) adsorption process was analyzed by performing kinetic experiments placed on a magnetic stirrer under controlled heating. The reached kinetic curves obtained for Ni(II) essays are plotted in Figure 8. The temperature-rising trend observed in this figure is very clear. Once the water was heated to 40 °C, the initially adsorbed Ni(II) desorbed, and the adsorption rate became negligible. The minimization of the Ni(II) attachment onto BC at higher temperatures demonstrates that the overall process of removal of this metal is an exothermic process.

The experimental results revealed that the adsorption reached in the first stages (mainly in the first minute of contact) at 50 °C was higher than the one achieved at 40 °C, while in both cases, a huge decay in the removal yield was found after longer contact times. This fact would be explained by the fact that at the beginning of the operation, BC was kept at room temperature and then added to the synthetic solution of Ni(II), which had already reached the evaluated temperature. In the first moments of the adsorption, the membrane is still heating up, meaning that the surface is colder than the bulk of the contaminated water. This interaction between the surface and the metal ions would still be similar to the one reached at 25 °C at the very beginning of the test. According to the results seen in the ID kinetic analysis (Figure 7a,b), there is an external mass transfer limitation as a rate-controlling step in the mass transfer of metal ions from the bulk to the adsorbent surface. This fact is also clear at the beginning of the treatment, as established by Inglezakis et al. [47], who indicated that the maximum difference of concentration of the adsorbate between the surface and the liquid occurs at this concrete moment. Thus, these authors associated the initial steps of the process with complete control of the adsorption rate by the external mass transfer, with negligible effect on the intraparticle diffusion [47]. With the adequate assumptions that could be taken into account at the very beginning of the process [47], his first-rate of adsorption could be simplified to first-order-like kinetics where the rate is proportional to a kinetic constant of film diffusion called k_f_ [47]. This kinetic constant would follow the Arrhenius equation (Equation (1)) while modifying temperature, being much faster at 50 °C. However, at long contact times, the adsorption–desorption equilibrium would rule the process. Looking at the results plotted in Figure 8, it is clear that the higher the temperature, the lower the removal of Ni(II), and, thus, the higher the desorption yield. At 25 °C, the equilibrium between adsorption and desorption is reached, but at temperatures higher or equal to 40 °C, the attached nickel starts desorbing due to the higher temperature reached inside the membrane (which at the equilibrium should be next to the one in the liquid). Therefore, the Ni(II) content in the liquid starts increasing until it reaches the same concentration as in the untreated solution. In the case of 40 °C, as the amount adsorbed was low, the time to reach this point is short, while at 50 °C, the time is longer; thus, the concentration after 1 min was ten times higher. Although a longer time is required until all nickel is released back to the bulk, in the end, in both experiments, a negligible removal was reached. 

**Figure 9:**
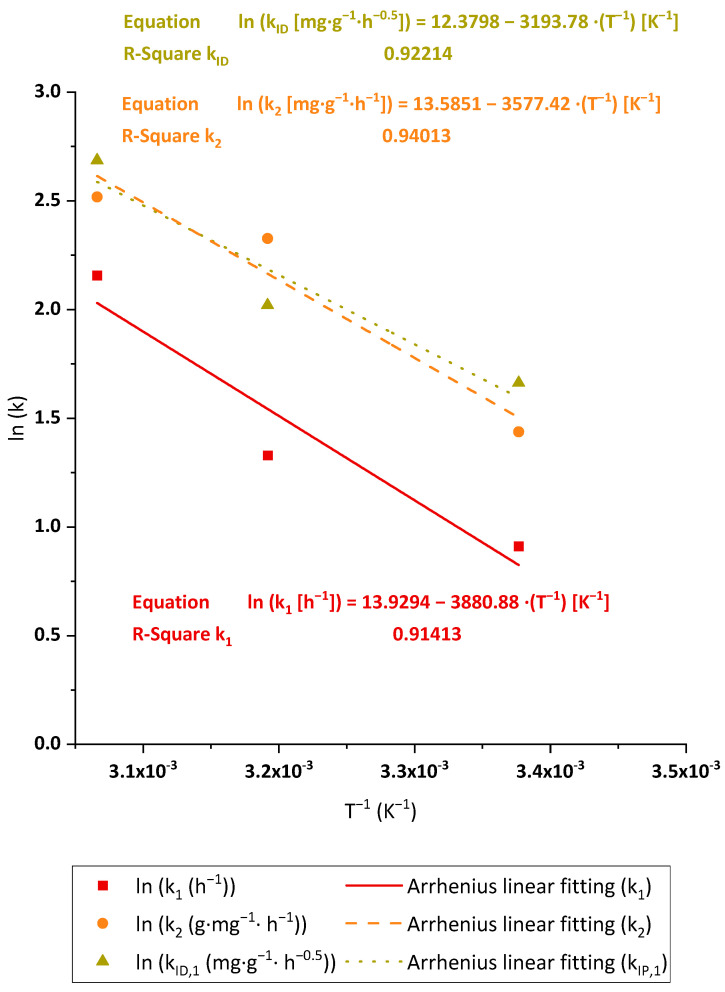
Arrhenius equation fitting of the Pb(II) adsorption kinetic constants of PFO (k_1_), PSO (k_2_) and first step ID (k_ID,1_). Tested conditions: 100 mL of sample volume, 1.5 mg·L^−1^ of initial metal concentration and pH 4.

The tests carried out for Pb(II) adsorption with BC under heating conditions proved the opposite behavior of the adsorbent surface with this contaminant. In this case, the experiments under 40 °C and 50 °C reached a slight adsorption capacity increase in comparison to 25 °C (20 and 23%, respectively), but the major impact was found in the rate of Pb(II) adsorption. The removal process became faster as the temperature rose. The effect was analyzed through the Arrhenius plot of the obtained PFO, PSO and ID constants obtained in the kinetic tests at the evaluated temperatures, whose resulting curves can be seen in Figure 9. The kinetic adjustments for Pb(II) adsorption onto BC at the different temperatures taken for the Arrhenius fitting are shown in Table S4. The linear fittings graphed in Figure 9 show three parallel lines. As the E_A_ is calculated from the slope of the linear adjustments, these results suggest that the energy value would be independent of the kinetic model selected for this purpose. As expected, the E_A_ values taken from the adjustments show low variation, between 29.75 and 32.27 kJ·mol^−1^. The mentioned values are relatively low, in the order of magnitude of the physisorption, fixed for process energies below 40 kJ·mol^−1^ in accordance with Inglezakis et al. [48].

#### 3.2.5. Isotherm Analysis

**Figure 10:**
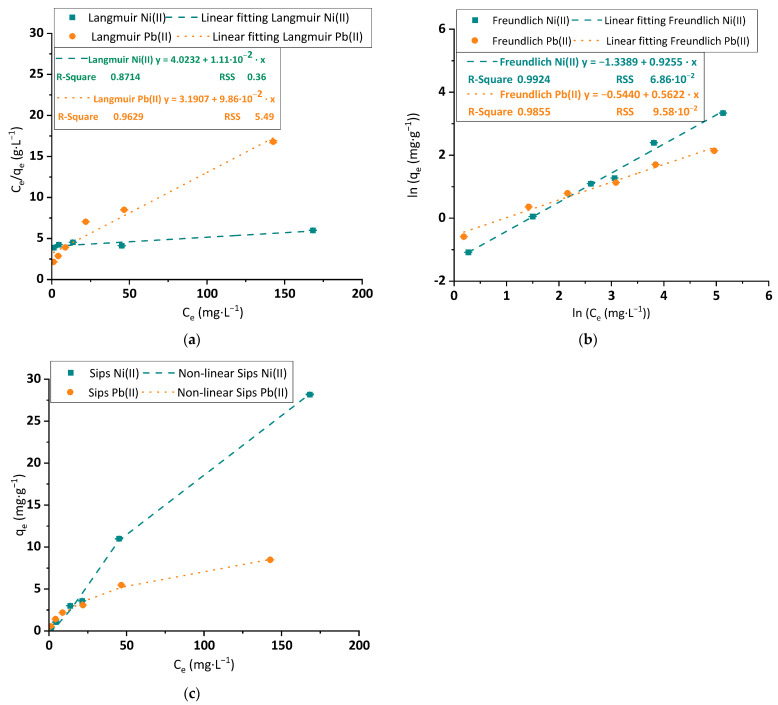
Linear fitting of (**a**) Langmuir; (**b**) Freundlich isotherm models; and (**c**) non-linear fitting to Sips isotherm model to experimental data of equilibrium adsorption capacity and concentration for Ni(II) (blue squares, fitting in blue dashed line) and Pb(II) (orange dots, fitting in orange dotted line) (1.5 mg·L^−1^ to 150 mg·L^−1^ for Pb(II) and 175 mg·L^−1^ for Ni(II)). Tested conditions: 25 mL of sample volume, 400 mg·L^−1^ of BC dosage and pH 4.

The equilibrium data were analyzed and fitted to a selection of isotherm models. The first adjusted models were the Langmuir, Freundlich and Sips equations, which usually represent the approach of the operation at the equilibrium with accuracy. The results of the linear adjustments of Langmuir and Freundlich and the non-linear adjustment of the Sips model are graphed in Figure 10a, Figure 10b and Figure 10c, respectively. The constants of each equation, obtained by the equations, are explained in Table 1. 

The curves seen in Figure 10a,b for both metals indicate that the linear fittings of Langmuir and Freundlich show low correlation parameters. Thus, the obtained values could only be used as the first approach to initialize a non-linear regression, which provides a more approximated result to the experimental data, whose R^2^ overpassed 0.99 in almost all the studied cases, as observed in Table 1. Nevertheless, the Sips model (Figure 10c), a model that supposes a curve growth shape similar to the Freundlich model and a saturation approach of the curve based on Langmuir equation, depicts the best simulation of the experimental data, especially in terms of RSS (several times smaller than the other analyzed models) for Ni(II) and Pb(II). The large value of the q_max_ for the application of the BC with the metals, especially the Langmuir q_max_ of Ni(II) over 100 mg·g^−1^, indicates a strong affinity between the tested material and both metals. These promising results would be the base for the future implementation of the BC membranes for wastewater treatment applications, and in the case of Ni(II), overpasses most of the previous Langmuir q_max_ results indicated in the bibliography for similar materials. 

Thanks to its accurate adjustment, the Sips model was selected as the best representation of the adsorption experimental data of these cations on the BC surface. Based on the wide differences between the values of the common parameters of Sips with Langmuir (q_max_) and Freundlich (k and n) models, the interpretation for the adsorption mechanism is an intermediate adsorption and intermediate surface properties between the monolayer occupation of active sites and smooth and homogeneously energetic-distributed surface explained by Langmuir, and the multilayer adsorption and rough and exponential distribution of active sites supposed by Freundlich. 

One aspect that must be evaluated in Figure 10c is that the shapes of Ni(II) and Pb(II) curves differ in the trend at large metal concentrations. Pb(II) isotherm seems closer to an exponential growth curve, typical of contaminants adsorbed from wastewater. Its shape would be classified as an L2 curve in accordance with Giles’ classification [49]. On the other hand, the linearity of the Ni(II) experimental curve is uncommon in the adsorption of wastewater treatment. Following the curve classification of Giles, this curve would be considered a C1 type. Previous authors found C-type curves in their adjustments and associated their results with weak interactions adsorbate-adsorbent, indicators of physisorption, that provided a proportional ratio of the solute between water and solid [50]. 

Another key consideration is the comparison between the maximum adsorption capacities measured in these equilibrium tests and the cationic demand of the BC, corresponding with the top adsorption capacity of cations by the material surface. While the BC cationic demand was up to 52.54 μeq·g^−1^, and the maximum removal of Ni(II) and Pb(II) were 960.32 and 81.98 μeq·g^−1^, respectively, the adsorptive removal of Ni(II) was only 5.47% and adsorptive removal of Pb(II) yield 64.09%. Thus, while Pb(II) is mainly removed by fixation of the cations to the BC surface, together with a third part of removal yield associated with other mechanisms, Ni(II) adsorption onto BC is negligible in its elimination mechanism and further studies must provide other alternatives to explain how this pollutant is separated by BC from water. 

#### 3.2.6. Adsorption Thermodynamics

**Figure 11:**
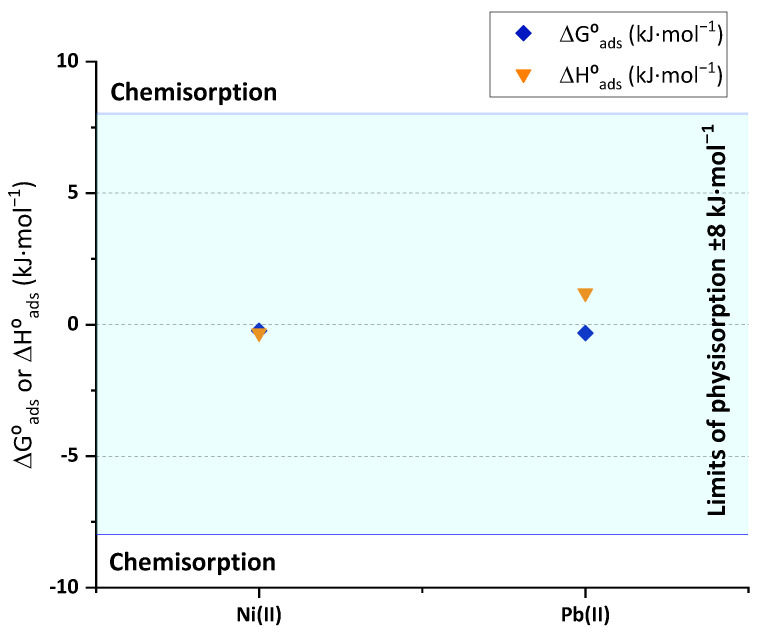
Heat of sorption (orange triangles) and mean free energy of Gibbs (blue diamonds) of Ni(II) and Pb(II) and the limit values for physisorption.

The adjustment of the isotherm experimental data to the models of Temkin and D-R provides the values of heat of sorption that are related to the enthalpy of adsorption and the mean free energy of adsorption, respectively. The resulting calculations of mean free energy of adsorption (ΔG°) and heat of sorption (ΔH°) of the Ni(II) and Pb(II) adsorption onto BC, as well as the interval correspondent to the limits of physical adsorption (between ±8 kJ·mol^−1^) are plotted on Figure 11. The parameters obtained from the non-linear adjustments of Temkin and D-R are listed in Table S5. The results in this figure confirm the expected behavior observed on the energy of activation calculated from Arrhenius. The observed heat and Gibbs’ energy of adsorption values fall within the limits of physisorption. In both cases, the Gibbs’ mean free energy is similar for both metals, and it is relatively low, between −200 and −300 J·mol^−1^. Other authors have published similarly low values of Gibbs’ energy of adsorption of Cd(II) onto chitosan/phosphorylated nanocellulose composites [51]. Together with the opposite enthalpy signs deduced by the temperature essays, the value of heat of sorption determined through Temkin isotherm of Pb(II) (1.20 kJ·mol^−1^) was an order of magnitude higher than the Ni(II) one (−0.32 kJ·mol^−1^). These results, combined with the totally different evolution of intraparticle diffusion model fittings, would mean that the removal mechanisms followed by each metal through BC application do not share the same pathways. The results of this thermodynamic analysis of low bonding energies between the surface and the metal ions could also be related to the results reached by Ni(II) adsorption onto BC under varied temperatures (Figure 8). Thanks to the reduced bonding energy and the exothermicity of the process, nickel was easily desorbed as the BC temperature was increased inside the hot water without the need for additional aid of reagents. These results would also provide a way to promote the selective adsorption or desorption of each metal through the adjustment of the influent temperature, which could be an interesting separation process. 

### 3.3. Mechanism Proposal for Ni(II) and Pb(II) Removal through BC Membranes 

**Figure 12:**
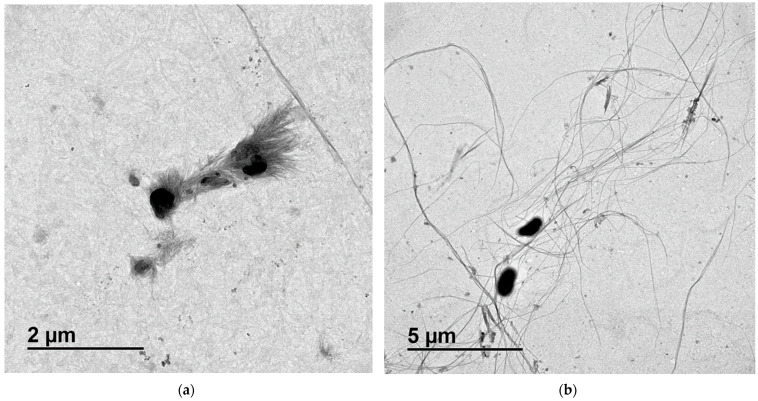
TEM images of BC after adsorption of (**a**) Ni(II) (150 mg·L^−1^) and (**b**) Pb(II) (175 mg·L^−1^). Tested conditions: 25 mL of sample volume, 400 mg·L^−1^ of BC dosage and pH 4.

The clear variations in the removal mechanisms of Ni(II) and Pb(II) observed along the analysis of the results of several tests require further studies about the surface of the material after adsorption. The TEM images of spent BC membranes after Ni(II) and Pb(II) adsorption are shown in Figure 12a and Figure 12b, respectively. Both images show relevant changes in the surface of BC membranes when compared to the initial raw material (Figure 2). 

The BC fibers and cell parts dispersed that were in contact with the synthetic solution of Ni(II) (Figure 12a) presented several crystals of small size, forming a network over the surface that connects different cell pieces or even BC fibers. The length of the crystals varied from a few nanometers to values close to the micrometer and their diameter was in the order of nanometers. 

In contrast, the BC fibers and cell parts applied to attract Pb(II) (Figure 12b) showed a darker color than the raw BC, meaning that the fibers are less transparent due to the deposition of this metal. The contaminant did not appear in the form of crystals but smoothly dispersed over the surface of the fibers. 

In addition, in comparison with the raw material, which presented a nanofiber network appearance, in these cases, the images showed single fibers and dispersed cells without strong connections. This fact could be associated with the stirring during the adsorption and the loss of interaction between fibers after suffering the coverage of metals. The fibers showed a few nanometers of diameter but microns in length, which is in accordance with the indicated parameters of most of the previous studies [52].

The observed TEM images allow for the interpretation of the experimental results in the isotherms. Once it is demonstrated that the adsorption of Ni(II) on the BC surface has a minor effect on the overall removal process, the crystallization process must be considered of main relevance. From the view of the authors, this result is remarkable. To the best of our knowledge, there are no previous publications covering Ni(II) removal through crystallization onto BC membranes. This process is ruled by the steps of nucleation, formation of critical nuclei and crystal growth [53]. The nucleation begins once a Ni^2+^ ion is attached to a BC active site. There, a change in the nickel species provides a first solid nucleus. Once the nuclei achieve a stable size (critical nuclei), they are able to grow into larger crystals by fixing more nickel from the bulk. Together with these results, it is relevant to say that the present study reveals the shortest time for equilibrium for Ni(II) adsorption (30 min), which suggests that crystallization onto smaller dosages of adsorbent would recover Ni(II) faster from contaminated solutions than larger dosages.

### 3.4. Advantages and Disadvantages of BC Implementation for Ni(II) and Pb(II) Treatment in Wastewater

The results obtained in this article are promising and reveal that BC could become a feasible and sustainable alternative for the treatment of heavy metal contamination present in wastewater. The synthetized BC membranes show a high affinity for the materials as they actively interact with various functional groups, as has been previously mentioned for other applications, as in the case of nanoparticle stabilizers [54]. Furthermore, BC allows the obtention of metal capacities much higher than those established by the surface active sites depicted by the cationic demand, especially in the case of Ni(II). This supposes an advance compared to other tested materials, whose maximum capacities are related to the maximum number of active sites. 

Another aspect to consider is the ease of implementation, as the membranes are simply added and removed after the batch operation without the need for complex operations. The reduced number of steps in their production and the fact that the material reached high metal capacities without any kind of surface modification minimizes the need for reagents in their synthesis, leading to a more sustainable production process. 

On the other hand, when compared to other nanocellulosic materials, BC has a lower cationic demand than, for example, catalytically oxidized cellulose nanofibers, which could surpass 1 meq·g^−1^ [55]. This issue mainly affects the efficiency while treating low concentrated waters, as the driving force to move ions from the bulk to the BC surface is reduced, and a large amount of surface anionic charges onto the surface would increase this interaction. This could be the cause for the need for dosages over 400 mg·L^−1^ of BC material to reach removal yields over 70% at low metal concentrations. 

The reasons for a low cationic demand value are, on the one hand, that the BC membranes did not suffer any surface treatment, while the nanofibers had an oxidation treatment to increase their number of surface anionic groups. On the other hand, the membrane-like shape of the BC promotes the adsorption onto the external surface of the membrane, while nanofibers are commonly used as hydrogels whose total contact surface is up to the sum of the external surface of nanofibers. In our case, the increase of the BC cationic demand could be performed without adding reagents by raising the surface area in contact with water. Addressing this matter, a study developed by Yuan et al. demonstrated that a sharp increase in the cationic demand is obtained by just disintegrating the membranes, reaching its maximum after beating suspensions with 10% of BC material at 8000 rpm [56]. This treatment after the membrane synthesis could provide an enhancement of the performance when treating waters with low metal content.

## 4. Conclusions

The adsorption process of Ni(II) and Pb(II) from water with BC membranes was carried out, reaching remarkable results in terms of metal adsorption and removal mechanism description. The characterization of the BC membranes showed that they presented a strong capability to attract cations, achieving 52.54 μeq·g^−1^ of cationic demand. The growth kinetics of the BC membrane were analyzed, which presupposes a novelty when compared to previous studies from the bibliography. This study revealed an efficient production time of the membranes of 4 days. TEM images of raw material showed that membranes were composed of complex BC fibers with nanosized diameters. A pH_zc_ of 6.30 indicated that the cationic attraction behavior of the BC surface occurred below this pH. The maximum removal efficiencies were achieved at 400 mg·L^−1^ of BC dosage at a pH of 4. The kinetic analysis indicated that the PSO kinetic model showed the highest correlation (R^2^ > 0.99) for both metals. The ID model showed a common decreasing slope trend for Pb(II) adsorption and a growth–plateau–growth tendency in the case of Ni(II) removal, which is not usually seen in the adsorption of this metal. The temperature effect on the kinetics revealed that Ni(II) removal was strongly exothermic, reaching the absence of activity over 40 °C, while Pb(II) removal was endothermic. The values of energy of activation of Pb(II) adsorption were in the order of physisorption processes. The Sips’ model showed the highest correlation parameter values for isotherm data for both metals. TEM images were taken to evaluate the surface morphology changes of spent BC with Pb(II) and Ni(II). The aspect of the fibers looked more disaggregated, and the metal coverage of the fibrils could be confirmed thanks to their opacity. The Ni(II) removal was mainly observed in the formation of numerous nanosized crystals. This result coincides with the value of maximum Ni(II) adsorption capacity, which was 20-fold higher than the cationic demand. On the other hand, Pb(II)-spent nanofibers were smoothly covered onto the BC surface. In general terms, this study provides detailed information that is useful for the further implementation of green and sustainable cellulosic nanomaterials to future applications in wastewater treatment as well as Ni(II) and Pb(II) recovery from contaminated solutions. To the best of our knowledge, this is the first study that demonstrates the removal of Ni(II) by adsorption and crystallization onto BC membranes, which opens the door for metal recovery as an insoluble species. 

## Figures and Tables

**Table 1:** Isotherm and non-linear correlation parameters obtained from the Langmuir, Freundlich and Sips models.

Model	Parameters [Units]	Values	
		Ni(II)	Pb(II)
Langmuir	k_L_ [L·mg^−1^]	2.13 × 10^−3^	3.09 × 10^−2^
	q_max,L_ [mg·g^−1^]	107.29	10.14
	R^2^	0.9971	0.9886
	RSS	3.69	1.41
Freundlich	k_F_ [mg^(1−1/n)^·L^(1/n)^·g^−1^]	1.08	0.58
	n_F_	0.26	1.78
	R^2^	0.9947	0.9933
	RSS	7.92	1.22
Sips	k_S_ [L^(1/nS)^·mol^−(1/nS)^]	1.49 × 10^−3^	2.73 × 10^−2^
	n_S_	0.69	1.50
	q_max,S_ [mg·g^−1^]	39.23	20.02
	R^2^	0.9989	0.9975
	RSS	1.45	0.28

## Supplementary Materials

The following supporting information can be downloaded at: https://www.mdpi.com/article/10.3390/polym15183684/s1, Table S1: Linear equations of the kinetic models; Table S2: Equations of the isotherm models; Table S3: Kinetic and correlation parameters of adjustment to PFO, PSO and Elovich models at room temperature for Ni(II) and Pb(II) batch adsorption onto BC membranes. Tested conditions: 100 mL of sample volume, 1.5 mg·L^−1^ of initial metal concentration and pH 4; Table S4: Kinetic and correlation parameters of adjustment to PFO, PSO and Elovich models at 40 °C and 50 °C for Pb(II) batch adsorption onto BC membranes. Tested conditions: 100 mL of sample volume, 1.5 mg·L^−1^ of initial metal concentration and pH 4; Table S5: Isotherm, thermodynamic and correlation parameters obtained from non-linear fitting of D-R and Temkin isotherm equations to Ni(II) and Pb(II) adsorption onto BC experimental equilibrium data. Tested conditions: 25 mL of sample volume, 400 mg·L^−1^ of BC dosage and pH 4.

## References

[1] Yang J., Li X., Xiong Z., Wang M., Liu Q. (2020). Environmental Pollution Effect Analysis of Lead Compounds in China Based on Life Cycle. Int. J. Environ. Res. Public. Health.

[2] Olabi A.G., Abbas Q., Shinde P.A., Abdelkareem M.A. (2023). Rechargeable batteries: Technological advancement, challenges, current and emerging applications. Energy.

[3] Gul T. (2023). Global EV Outlook 2023.

[4] Zaynab M., Al-Yahyai R., Ameen A., Sharif Y., Ali L., Fatima M., Khan K.A., Li S. (2022). Health and environmental effects of heavy metals. J. King Saud Univ. Sci..

[5] Muñoz P.Q. (2015). Hazardous Substances and Limits of Discharge for Indirect Dumpings in Autonomic Legislation.

[6] Kumar V., Dwivedi S.K. (2021). A review on accessible techniques for removal of hexavalent Chromium and divalent Nickel from industrial wastewater: Recent research and future outlook. J. Clean. Prod..

[7] Kumar V., Dwivedi S.K., Oh S. (2022). A critical review on lead removal from industrial wastewater: Recent advances and future outlook. J. Water Process Eng..

[8] Chai W.S., Cheun J.Y., Kumar P.S., Mubashir M., Majeed Z., Banat F., Ho S.-H., Show P.L. (2021). A review on conventional and novel materials towards heavy metal adsorption in wastewater treatment application. J. Clean. Prod..

[9] Qiao A., Cui M., Huang R., Ding G., Qi W., He Z., Klemeš J.J., Su R. (2021). Advances in nanocellulose-based materials as adsorbents of heavy metals and dyes. Carbohydr. Polym..

[10] Minceva M., Fajgar R., Markovska L., Meshko V. (2008). Comparative Study of Zn^2+^, Cd^2+^, and Pb^2+^ Removal from Water Solution Using Natural Clinoptilolitic Zeolite and Commercial Granulated Activated Carbon. Equilibrium of Adsorption. Sep. Sci. Technol..

[11] Barakat M.A. (2011). New trends in removing heavy metals from industrial wastewater. Arab. J. Chem..

[12] Varghese A.G., Paul S.A., Latha M.S. (2019). Remediation of heavy metals and dyes from wastewater using cellulose-based adsorbents. Environ. Chem. Lett..

[13] Sridevi M., Nirmala C., Jawahar N., Arthi G., Vallinayagam S., Sharma V.K. (2021). Role of nanomaterial’s as adsorbent for heterogeneous reaction in waste water treatment. J. Mol. Struct..

[14] Reshmy R., Philip E., Madhavan A., Pugazhendhi A., Sindhu R., Sirohi R., Awasthi M.K., Pandey A., Binod P. (2022). Nanocellulose as green material for remediation of hazardous heavy metal contaminants. J. Hazard. Mater..

[15] Li A., Xu D., Luo L., Zhou Y., Yan W., Leng X., Dai D., Zhou Y., Ahmad H., Rao J. (2021). Overview of nanocellulose as additives in paper processing and paper products. Nanotechnol. Rev..

[16] Faiz Norrrahim M.N., Mohd Kasim N.A., Knight V.F., Mohamad Misenan M.S., Janudin N., Ahmad Shah N.A., Kasim N., Wan Yusoff W.Y., Mohd Noor S.A., Jamal S.H. (2021). Nanocellulose: A bioadsorbent for chemical contaminant remediation. RSC Adv..

[17] Grishkewich N., Mohammed N., Tang J., Tam K.C. (2017). Recent advances in the application of cellulose nanocrystals. Curr. Opin. Colloid Interface Sci..

[18] Campano C., Balea A., Blanco A., Negro C. (2016). Enhancement of the fermentation process and properties of bacterial cellulose: A review. Cellulose.

[19] Chawla P., Bajaj I., Survase S., Singhal R. (2009). Microbial Cellulose: Fermentative Production and Applications. Food Technol. Biotechnol..

[20] Mohite B.V., Patil S.V. (2014). A novel biomaterial: Bacterial cellulose and its new era applications. Biotechnol. Appl. Biochem..

[21] Zhong C. (2020). Industrial-Scale Production and Applications of Bacterial Cellulose. Front. Bioeng. Biotechnol..

[22] Torres F.G., Arroyo J.J., Troncoso O.P. (2019). Bacterial cellulose nanocomposites: An all-nano type of material. Mater. Sci. Eng. C.

[23] Ashjaran A., Yazdanshenas M.E., Rashidi A., Khajavi R., Rezaee A. (2013). Overview of bio nanofabric from bacterial cellulose. J. Text. Inst..

[24] Chen S., Zou Y., Yan Z., Shen W., Shi S., Zhang X., Wang H. (2009). Carboxymethylated-bacterial cellulose for copper and lead ion removal. J. Hazard. Mater..

[25] Jin X., Xiang Z., Liu Q., Chen Y., Lu F. (2017). Polyethyleneimine-bacterial cellulose bioadsorbent for effective removal of copper and lead ions from aqueous solution. Bioresour. Technol..

[26] Shen W., Chen S.Y., Shi S.K., Li X., Zhang X., Hu W.L., Wang H.P. (2009). Adsorption of Cu(II) and Pb(II) onto diethylenetriamine-bacterial cellulose. Carbohydr. Polym..

[27] Chen S.Y., Shen W., Yu F., Hu W.L., Wang H.P. (2010). Preparation of Amidoximated Bacterial Cellulose and Its Adsorption Mechanism for Cu^2+^ and Pb^2+^. J. Appl. Polym. Sci..

[28] Mohite B.V., Patil S.V. (2014). Bacterial cellulose of Gluconoacetobacter hansenii as a potential bioadsorption agent for its green environment applications. J. Biomater. Sci. Polym. Ed..

[29] Santos S., Carbajo J., Villar J. (2013). The Effect of Carbon and Nitrogen Sources on Bacterial Cellulose Production and Properties from Gluconacetobacter sucrofermentans CECT 7291 Focused on its use in Degraded Paper Restoration. Bioresources.

[30] Santos S.M., Carbajo J.M., Gómez N., Ladero M., Villar J.C. (2017). Modification of Bacterial Cellulose Biofilms with Xylan Polyelectrolytes. Bioengineering.

[31] Seniya D., Verma S., Trivedia S., Verma R., Vijayarti H.S., Vyas S. (2012). Metal Stress and Antibiotic Susceptibility Profileof Some Bacterial and Fungal Strains. J. Pure Appl. Microbiol..

[32] Balea A., Sanchez-Salvador J.L., Monte M.C., Merayo N., Negro C., Blanco A. (2019). In Situ Production and Application of Cellulose Nanofibers to Improve Recycled Paper Production. Molecules.

[33] Hosseini Talari M., Tabrizi N.S., Babaeipour V., Halek F. (2022). Adsorptive removal of organic pollutants from water by carbon fiber aerogel derived from bacterial cellulose. J. Sol-Gel Sci. Technol..

[34] Ojembarrena F.d.B., Sammaraie H., Campano C., Blanco A., Merayo N., Negro C. (2022). Hexavalent Chromium Removal from Industrial Wastewater by Adsorption and Reduction onto Cationic Cellulose Nanocrystals. Nanomaterials.

[35] (2017). Deutsche Einheitsverfahren zur Wasser-, Abwasser- und Schlammuntersuchung–Allgemeine Angaben (Gruppe A)–Teil 51: Kalibrierung von Analysenverfahren–Lineare Kalibrierfunktion (A 51).

[36] (2008). Chemische Analytik—Nachweis-, Erfassungs- und Bestimmungsgrenze unter Wiederholbedingungen—Begriffe, Verfahren, Auswertung.

[37] (1994). Accuracy (trueness and precision) of measurement methods and results—Part 1: General principles and definitions.

[38] Ojembarrena F.d.B., Sánchez-Salvador J.L., Mateo S., Balea A., Blanco A., Merayo N., Negro C. (2022). Modeling of Hexavalent Chromium Removal with Hydrophobically Modified Cellulose Nanofibers. Polymers.

[39] Budhiono A., Rosidi B., Taher H., Iguchi M. (1999). Kinetic aspects of bacterial cellulose formation in nata-de-coco culture system. Carbohydr. Polym..

[40] Usawattanakul N., Torgbo S., Sukyai P., Khantayanuwong S., Puangsin B., Srichola P. (2021). Development of Nanocomposite Film Comprising of Polyvinyl Alcohol (PVA) Incorporated with Bacterial Cellulose Nanocrystals and Magnetite Nanoparticles. Polymers.

[41] Campano C., Merayo N., Negro C., Blanco A. (2018). In situ production of bacterial cellulose to economically improve recycled paper properties. Int. J. Biol. Macromol..

[42] Attar K., Demey H., Bouazza D., Sastre A.M. (2019). Sorption and Desorption Studies of Pb(II) and Ni(II) from Aqueous Solutions by a New Composite Based on Alginate and Magadiite Materials. Polymers.

[43] Putro J., Kurniawan A., Ismadji S., Ju Y.-H. (2017). Nanocellulose based biosorbents for wastewater treatment: Study of isotherm, kinetic, thermodynamic and reusability. Environ. Nanotechnol. Monit. Manag..

[44] Dotto G.L., Pinto L.A.A. (2011). Adsorption of food dyes acid blue 9 and food yellow 3 onto chitosan: Stirring rate effect in kinetics and mechanism. J. Hazard. Mater..

[45] Wu F.-C., Tseng R.-L., Juang R.-S. (2009). Initial behavior of intraparticle diffusion model used in the description of adsorption kinetics. Chem. Eng. J..

[46] Miller A., Wildeman T., Figueroa L. (2013). Zinc and nickel removal in limestone based treatment of acid mine drainage: The relative role of adsorption and co-precipitation. Appl. Geochem..

[47] Inglezakis V.J., Balsamo M., Montagnaro F. (2020). Liquid–Solid Mass Transfer in Adsorption Systems—An Overlooked Resistance?. Ind. Eng. Chem. Res..

[48] Inglezakis V.J., Zorpas A.A. (2012). Heat of adsorption, adsorption energy and activation energy in adsorption and ion exchange systems. Desalin Water Treat..

[49] Gauden P., Terzyk A.P., Kowalczyk P., Aranovich G.L., Ćwiertnia M., Furmaniak S., Rychlicki G. (2008). Giles’ classification of solute adsorption isotherms for binary non-electrolyte solutions via lattice DFT supported by experimental sorption data from aqueous solutions on carbonaceous materials. Carbon Materials: Theory and Practice.

[50] Lawrence M.A.M., Davies N.A., Edwards P.A., Taylor M.G., Simkiss K. (2000). Can adsorption isotherms predict sediment bioavailability?. Chemosphere.

[51] Brandes R., Belosinschi D., Brouillette F., Chabot B. (2019). A new electrospun chitosan/phosphorylated nanocellulose biosorbent for the removal of cadmium ions from aqueous solutions. J. Environ. Chem. Eng..

[52] Iqbal D., Zhao Y., Zhao R., Russell S.J., Ning X. (2022). A Review on Nanocellulose and Superhydrophobic Features for Advanced Water Treatment. Polymers.

[53] Patel D.D., Anderson B.D. (2013). Maintenance of supersaturation II: Indomethacin crystal growth kinetics versus degree of supersaturation. J. Pharm. Sci..

[54] Vinogradov M.I., Makarov I.S., Golova L.K., Gromovykh P.S., Kulichikhin V.G. (2020). Rheological Properties of Aqueous Dispersions of Bacterial Cellulose. Processes.

[55] Sanchez-Salvador J.L., Campano C., Negro C., Monte M.C., Blanco A. (2021). Increasing the Possibilities of TEMPO-Mediated Oxidation in the Production of Cellulose Nanofibers by Reducing the Reaction Time and Reusing the Reaction Medium. Adv. Sustain. Syst..

[56] Yuan J., Wang T., Huang X., Wei W. (2016). Dispersion and beating of bacterial cellulose and their influence on paper properties. BioResources.

