# Supplementary material for: Ni(II) and Pb(II) Removal Using Bacterial Cellulose Membranes
Source: Polymers (Basel). 2023 Sep 7;15(18):3684. doi: 10.3390/polym15183684 (PMC10534930; doi:10.3390/polym15183684)
Supplement: Supplementary file 1 [file polymers-15-03684-s001.zip › polymers-2552719-supplementary.pdf]

Supplementary Materials:

Table S1. Linear equations of the kinetic models

Model	Equations		Ref.
PFO	$\ln(q_e - q) - \ln(q_e) = -k_1 \cdot t$	(S1)	[39]
PSO	$\frac{t}{q} = \left(\frac{1}{q_e^2 \cdot k_2}\right) + \left(\frac{1}{q_e}\right) \cdot t$	(S2)	[40]
Elovich	$q = \frac{1}{\beta} \cdot \ln(t) + \frac{1}{\beta}\ln(\alpha \cdot \beta)$	(S3)	[41]
ID	$q = k_i \cdot t^{0.5} + C$	(S4)	[42]

Table S2. Equations of the isotherm models

Model	Equations		Ref.
Langmuir	$\frac{C_e}{q_e} = \frac{1}{K_L \cdot q_{max}} + \left(\frac{1}{q_{max}}\right) \cdot C_e$	(S5)	[43]
Freundlich	$\ln(q_e) = \frac{1}{n_F} \cdot \ln(C_e) + \ln(K_F)$	(S6)	
Temkin	$q_e = B_T \cdot \ln(A_T) + B_T \cdot \ln(C_e)$	(S7)	
	$B_T = \frac{R \cdot T}{b_T}$	(S8)	
D-R	$\ln(q_e) = \ln(q_{max}) - B_{DR} \cdot \varepsilon^2$	(S9)	
	$\varepsilon = R \cdot T \cdot \ln\left(1 + \frac{1}{C_e}\right)$	(S10)	
	$E_{DR} = \frac{1}{\sqrt{2 \cdot B_{DR}}}$	(S11)	
Sips	$q_e = \frac{K_S \cdot q_{max} \cdot C_e^{1/n_S}}{1 + K_S \cdot C_e^{1/n_S}}$	(S12)	

Table S3. Kinetic and correlation parameters of adjustment to PFO, PSO and Elovich models at room temperature for Ni(II) and Pb(II) batch adsorption onto BC membranes. Tested conditions: 100 mL of sample volume, 1.5 $mg \cdot L^{-1}$ of initial metal concentration and pH 4

Kinetic Model	Metal		Ni(II)	Pb(II)
PFO	Kinetic parameters	k_1 [h^{-1}]	14.05	13.44
		q_e [$mg \cdot g^{-1}$]	0.37	1.73
	Correlation parameters	R^2	0.9384	0.9598
		RSS	$1.74 \cdot 10^{-2}$	0.28
PSO	Kinetic parameters	k_2 ($mg \cdot g^{-1} \cdot h^{-1}$)	8.98	8.60
		q_e ($mg \cdot g^{-1}$)	0.46	1.88
	Correlation parameters	R^2	0.9484	0.9826
		RSS	$1.70 \cdot 10^{-3}$	0.12
Elovich	Kinetic parameters	α [$h \cdot mg \cdot g^{-1}$]	67.33	120.56
		β [$g \cdot mg^{-1}$]	19.66	3.50
	Correlation parameters	R^2	0.9860	0.9885
		RSS	$1.50 \cdot 10^{-3}$	$8.21 \cdot 10^{-2}$

Table S4. Kinetic and correlation parameters of adjustment to PFO, PSO and Elovich models at 40 °C and 50 °C for Pb(II) batch adsorption onto BC membranes. Tested conditions: 100 mL of sample volume, 1.5 $mg \cdot L^{-1}$ of initial metal concentration and pH 4

Kinetic Model		Parameter [units]	40 °C	50 °C
PFO	Kinetic parameters	k_1 [h^{-1}]	12.93	116.20
		q_e [h^{-1}]	2.38	2.21
	Correlation parameters	R^2	0.9980	0.9906
		RSS	$2.18 \cdot 10^{-2}$	0.14
PSO	Kinetic parameters	k_2 [$mg \cdot g^{-1} \cdot h^{-1}$]	7.07	116.80
		q_e [$mg \cdot g^{-1}$]	2.58	2.26
	Correlation parameters	R^2	0.9966	0.9931
		RSS	$6.64 \cdot 10^{-2}$	$9.88 \cdot 10^{-2}$
ID	Kinetic parameters	$k_{IP,1}$ [$mg \cdot h^{-1} \cdot h^{-0.5}$]	7.54	14.68
		C [$mg \cdot g^{-1}$]	-0.55	0
	Correlation parameters	R^2	1	1

Table S5. Isotherm, thermodynamic and correlation parameters obtained from non-linear fitting of D-R and Temkin isotherm equations to Ni(II) and Pb(II) adsorption onto BC experimental equilibrium data. Tested conditions: 25 mL of sample volume, 400 $mg \cdot L^{-1}$ of BC dosage and pH 4

Model	Parameters		Ni(II)	Pb(II)
D-R	Isotherm parameters	B_{DR} [$mol^2 \cdot J^{-2}$]	$9.49 \cdot 10^{-6}$	$4.94 \cdot 10^{-6}$
		q_{max} [$mg \cdot g^{-1}$]	8.73	4.83
	Thermodynamic parameters	E_{DR} [$J \cdot mol^{-1}$]	229.52	318.22
	Correlation parameters	R^2	0.6325	0.7868
		RSS	415.75	17.94
Temkin	Isotherm parameters	A_T [$L \cdot g^{-1}$]	0.14	0.36
		b_T [$J \cdot mol^{-1}$]	316.60	1202.91
	Correlation parameters	R^2	0.8586	0.9522
		RSS	253.72	6.9219
